# Baseline 18F-FDG PET/CT metabolic parameters and systemic inflammatory markers in advanced NSCLC treated with first-line immunotherapy: an exploratory retrospective analysis

**DOI:** 10.3389/fonc.2026.1837219

**Published:** 2026-08-05

**Authors:** Blerina Resuli, Julia Walter, Friederike Völter, Diego Kauffmann-Guerrero, Paul Dahlmann, Raphael Hirner, Lena Unterrainer, Pontus Mertsch, Paola Arnold, Paula Mras, Gabriela Leuschner, Johannes Rübenthaler, Christian Schneider, Jürgen Behr, Amanda Tufman

**Affiliations:** 1 Department of Medicine V, LMU University Hospital of Munich, Munich, Germany; 2 Comprehensive Pneumology Center Munich (CPC-M), German Center for Lung Research (DZL), and Bavarian Cancer Research Center (BZKF), Munich, Germany; 3 Department of Nuclear Medicine, LMU University Hospital, Munich, Germany; 4 Department of Radiology, LMU University Hospital, Munich, Germany; 5 Department of Thoracic Surgery, Ludwig-Maximilian-University (LMU) University Hospital Munich, Munich, Germany

**Keywords:** 18F-FDG PET/CT, inflammatory marker, MTV, NSCLC, SUV

## Abstract

**Background:**

Predictive biomarkers for patients with advanced non-small cell lung cancer (NSCLC) treated with immune checkpoint inhibitors (ICIs) remain limited. Systemic inflammation plays a crucial role in cancer progression and immune regulation. We evaluated whether baseline 18F-FDG PET/CT metabolic parameters and systemic inflammatory markers are associated with treatment response and survival in patients with advanced NSCLC treated with first-line ICIs.

**Methods:**

Among 142 patients treated with first-line ICIs either as monotherapy or combined with chemotherapy, between October 2018 and December 2023, 116 had baseline PET/CT imaging. Quantitative analyses of standardized uptake value max (SUVmax) and metabolic tumor volume (MTV) were restricted to 71 patients whose scans were performed at our institution. Associations with response, progression free survival (PFS), and overall survival (OS) were analyzed.

**Results:**

In multivariable models including clinical variables, PET biomarkers and inflammatory markers, no individual PET or inflammatory biomarker remained independently associated with survival. Combined models suggested better prognostic discrimination compared with PET-only or inflammation-only models (OS C-index 0.745; PFS C-index 0.750). In contrast, sex, TTF-1 status, and the presence of adrenal and lung metastases emerged as independent predictors of OS and/or PFS. SUVmax correlated significantly with baseline CRP levels, while no significant associations were observed with LDH or NLR. MTV was associated with LDH but not with CRP or NLR.

**Conclusions:**

Baseline PET-derived metabolic parameters and systemic inflammatory biomarkers may provide complementary prognostic information in advanced NSCLC treated with first-line ICIs. Integrated clinical, metabolic, and inflammatory models showed improved prognostic performance, supporting further prospective validation of multimodal risk stratification strategies.

## 1 Background

Non-small cell lung cancer (NSCLC) is a biologically and clinically heterogeneous disease, characterized by substantial variability in tumor genetics, histology, metabolic activity, and immune microenvironment, all of which influence treatment response and prognosis (1, 2). Despite major therapeutic advances, including the widespread use of immune checkpoint inhibitors (ICIs), reliable biomarkers to predict treatment response and survival outcomes remain limited (2).

ICIs targeting programmed cell death protein 1 (PD-1) or its ligand PD-L1 have become standard of care in the first-line treatment of advanced or metastatic NSCLC without actionable driver mutations, either as monotherapy or in combination with platinum-based chemotherapy (3–6). However, only a subset of patients achieves durable clinical benefit, while others experience early disease progression, highlighting the need for improved patient stratification.

18F-fluorodeoxyglucose positron emission tomography/computed tomography (18F-FDG PET/CT) is routinely used for staging and response assessment in NSCLC. Beyond its diagnostic role, PET-derived metabolic parameters such as maximum standardized uptake value (SUVmax), metabolic tumor volume (MTV), and total lesion glycolysis (TLG) have been investigated as potential biomarkers of tumor aggressiveness and prognosis (7–10). High metabolic tumor burden has been associated with unfavorable outcomes in several malignancies, although data in immunotherapy-treated NSCLC remain inconsistent.

Systemic inflammation plays a crucial role in cancer progression and immune regulation. Inflammatory biomarkers such as C-reactive protein (CRP), lactate dehydrogenase (LDH), and the neutrophil-to-lymphocyte ratio (NLR) have been associated with prognosis and response to ICIs in NSCLC (11–14). However, the relationship between baseline inflammatory status and tumor metabolic activity assessed by 18F-FDG PET/CT has not been fully elucidated.

The aim of this study was to explore the prognostic and predictive association between baseline tumor metabolic activity assessed by 18F-FDG PET/CT and systemic inflammatory status, and to evaluate their relationship with treatment response and survival in patients with advanced NSCLC undergoing first-line immune checkpoint inhibition.

## 2 Methods

### 2.1 Study design and patients

This retrospective single-center study was conducted at the Department of Medicine V, Ludwig-Maximilians University (LMU) Hospital, Munich. Clinical records and tumor board decisions were systematically reviewed for all consecutive patients who fulfilled the inclusion criteria. Eligible patients were required to have a histologically confirmed diagnosis of NSCLC between October 2018 and December 2023, baseline 18F-FDG PET/CT performed for staging prior to initiation of systemic therapy, and an age of 18 years or older at diagnosis. Further inclusion criteria were written informed consent for the use of clinical data for research purposes, indication for first-line ICIs therapy according to current guidelines (either as monotherapy or in combination with platinum-based chemotherapy depending on PD-L1 expression), Eastern Cooperative Oncology Group (ECOG) performance status of 0–2, no prior systemic therapy for advanced NSCLC, and stage IV or stage III disease not suitable for local treatment according to the eighth edition of the American Joint Committee on Cancer (AJCC)/International Association for the Study of Lung Cancer (IASCL) TNM staging System.

Exclusion criteria were clinical or biological contraindications to ICI therapy, blood glucose >9 mmol/L at the time of PET/CT, or a delay exceeding three months between baseline PET/CT and treatment initiation. The study was conducted in accordance with the Declaration of Helsinki and approved by the Institutional Ethics Committee of the LMU, (Approval-code: 476-16 UE, approval date 5 August 2016). The requirement for informed consent was waived by the institutional ethics committee due to the retrospective nature of the study and the use of anonymized clinical data. **This study is reported in accordance with the Strengthening the Reporting of Observational Studies in Epidemiology (STROBE) reporting guideline.**

### 2.2 Baseline characteristics

Baseline clinical, pathological, and laboratory characteristics were retrieved from the electronic medical records for all eligible patients. Demographic variables included age at diagnosis, expressed as mean, standard deviation, and range, and sex distribution (male vs. female). Smoking history was categorized as current, former, or never smoker, whenever this information was available.

The histopathological subtype of NSCLC was documented according to World Health Organization (WHO) classification and included adenocarcinoma, squamous cell carcinoma, large cell carcinoma, and adenosquamous carcinoma. In addition, PD-L1 expression was reported where available, stratified by tumor proportion score (Tumor Proportion Score (TPS)<1%, 1–49%, and ≥50%) and thyroid Transcription Factor-1 (TTF-1) status was also recorded for adenocarcinoma patients.

Tumor stage was defined using the 8th edition of the AJCC TNM classification. Information on sites of metastatic involvement at baseline was systematically collected, including brain, bone, liver, adrenal glands, pleural effusion, and contralateral lung metastases. The number of metastatic sites was also categorized (<3 vs. ≥3), a commonly used threshold to distinguish limited versus more extensive metastatic burden and to facilitate clinically interpretable stratification. Performance status was assessed using the ECOG scale, ranging from 0 to 2.

Patients were classified into ICI monotherapy and chemo-immunotherapy according to first-line treatment.

Laboratory data obtained closest to treatment initiation were collected as indicators of systemic inflammation and tumor burden. These included serum LDH, CRP, and the NLR, derived from routine blood counts. LDH and CRP were recorded as continuous variables, while NLR was analyzed both as a continuous parameter and as a categorical variable using clinically relevant cutoffs.

Finally, treatment allocation was documented, distinguishing patients receiving first-line ICIs monotherapy from those receiving combination ICIs with platinum-based chemotherapy, according to PD-L1 expression and guideline-based treatment decisions.

### 2.3 Imaging protocol and analysis

All patients fasted for 6 hours before tracer injection. Blood glucose levels were confirmed to be below 150 mg/dL at the time of 18F-FDG administration. PET/CT scans were acquired during mid-breath-hold. The SUV was calculated using the following standard formula:


$$\mathrm{SUV}=\frac{\mathrm{Sphere}\;\mathrm{activity}\left[\frac{\mathrm{Bg}}{\mathrm{ml}}\right]\;*\mathrm{body}\;\mathrm{weight}(\mathrm{kg})}{\mathrm{injected}\;\mathrm{dose}}$$

PET/CT images were analyzed with Hermia Affinity version 4.0.1 (Hermes Medical Solutions, Stockholm, Sweden). SUVmax was defined as the maximum voxel value within the tumor region of interest. MTV was delineated semi-automatically using a fixed threshold of 42% of SUVmax, with physiologic tracer uptake excluded from the contour. The 42% threshold was selected because it represents one of the most widely used and validated segmentation approaches for FDG PET volumetric analysis in NSCLC and allows comparability with previous studies evaluating MTV in immunotherapy-treated patients.

Importantly, MTV analysis was performed only in patients whose baseline PET/CT scans were acquired at our institution, since external imaging data lacked the standardization required for volumetric calculation. As a result, the MTV cohort comprised a subset of the overall study population. To explore potential selection bias related to this restriction, baseline characteristics of patients with and without available MTV measurements were compared and are presented in **Supplementary Table 1**.

SUVmax categories (<10, 10–14, ≥15) were selected based on clinically interpretable metabolic activity strata and in line with previously published PET/CT studies in NSCLC that have used similar threshold-based approaches to facilitate clinical interpretation and comparison across cohorts (7–10, 15, 16).

MTV categories were empirically derived from the observed distribution of values within the study population (≤110, 111–290, 291–606, and >606) to enable exploratory stratified analyses of tumor burden, given the absence of universally accepted clinical thresholds for MTV in immunotherapy-treated NSCLC.

This approach is consistent with prior exploratory studies evaluating PET-derived biomarkers in NSCLC, where categorization is frequently applied to facilitate clinical interpretation and comparison across studies (7–10, 15, 16).

Formal reproducibility analyses of MTV segmentation were not performed because of the retrospective nature of the study. Scanner-specific reconstruction parameters, EARL accreditation status, and formal inter-reader reproducibility assessments were not available in the retrospectively collected dataset and therefore could not be reported.

### 2.4 Statistical analysis

Continuous variables were expressed as mean ± standard deviation (SD), while categorical variables were reported as absolute counts and percentages. Between-group comparisons were performed using the Student’s t-test for continuous variables and the Chi-squared test for categorical variables.

Univariate correlations between PET-derived parameters and inflammatory biomarkers were assessed using Spearman’s rank correlation coefficient. To control for potential confounding factors, linear regression models were constructed for LDH, CRP, and NLR with SUVmax as the main predictor, adjusted for age, sex, smoking history, number of metastatic sites, and TTF-1 expression.

Survival outcomes were defined as overall survival (OS), measured from initiation of systemic therapy until death from any cause, and progression-free survival (PFS), measured from initiation of therapy until documented disease progression or death. Kaplan–Meier survival curves were generated to estimate OS and PFS, with differences compared using the log-rank test. Multivariate Cox proportional hazards regression models were then used to evaluate the prognostic value of PET-derived parameters and clinical variables, including sex, age, smoking status, metastatic sites, PD-L1 expression, and TTF-1 status. Additional multivariable Cox regression models were constructed incorporating PET biomarkers (SUVmax and MTV), inflammatory biomarkers (CRP, LDH and NLR), and clinical covariates within the same models. Continuous log-transformed variables were used for SUVmax, MTV, CRP, LDH and NLR. Continuous log-transformed PET biomarkers were used as the primary variables for all multivariable prognostic analyses in order to preserve statistical information and avoid potential bias associated with categorization. Restricted cubic spline models were fitted to investigate potential nonlinear associations. To minimize the risk of overfitting, primary multivariable models were restricted to a limited number of clinically relevant covariates selected *a priori* based on established prognostic relevance and biological plausibility.

Model discrimination was evaluated using Harrell’s concordance index (C-index), Akaike Information Criterion (AIC), and Bayesian Information Criterion (BIC). Proportional hazards assumptions were assessed using Schoenfeld residuals. False discovery rate correction according to Benjamini-Hochberg was applied to exploratory biomarker and interaction analyses. Missing data were handled using complete-case analysis because missingness was low (<5% for all variables). The extent of missing data for each study variable is reported in **Supplementary Table 6**. Penalized Cox regression using LASSO regularization was performed as a sensitivity analysis to evaluate model stability. Internal validation was additionally performed using bootstrap resampling.

Due to the limited number of events, we predefined one primary multivariable Cox model including log-transformed SUVmax, log-transformed MTV, age, sex, PD-L1 expression and treatment group. The number of observed events was 39 for OS and 54 for PFS. The corresponding events-per-variable were 6.17 for OS and 8.50 for PFS. Additional extended and stratified models were considered exploratory and are presented in the Supplementary Material. Extended models including TTF-1 expression and metastatic burden variables were considered exploratory because of the limited events-per-variable ratio and are therefore presented exclusively in the Supplementary Material as hypothesis-generating analyses. Treatment group (ICI monotherapy versus chemo-immunotherapy) was included as an adjustment variable in all predefined multivariable analyses to account for treatment heterogeneity.

All statistical tests were two-sided, and a p-value<0.05 was considered statistically significant. Analyses were performed using R Studio (version 4.0), and figures and tables were created using R Studio and Microsoft Excel. Treatment response was assessed according to Response Evaluation Criteria in Solid Tumors (RECIST) version 1.1.

## 3 Results

### 3.1 Patient characteristics

Among 142 patients with advanced NSCLC treated between October 2018 and December 2023, baseline 18F-FDG PET/CT imaging was available for 116 patients. Quantitative volumetric analyses (SUVmax and MTV) analyses were restricted to the 71 patients whose baseline imaging had been acquired at our institution to ensure methodological consistency and comparability of measurements. A comparison of baseline characteristics between patients with and without available MTV measurements is provided in **Supplementary Table 1**. Given the small number of patients without MTV data, these analyses should be interpreted cautiously.

Based on the AJCC 8th edition classification, most patients had stage IV disease at diagnosis (n = 113, 98%), whereas only three patients (2%) had stage III disease considered unsuitable for definitive local therapy.

Baseline clinical and pathological characteristics stratified by SUV categories (<10, 10–14, and ≥15) are summarized in **Table 1**. Age at diagnosis did not differ significantly between groups (mean 62.6, 61.7, and 66.8 years, respectively; p = 0.26). Sex distribution was comparable across SUV strata, with males accounting for 66.7%, 62.5%, and 42.3% of patients in the three groups (p = 0.20).

**Table 1:** Patient characteristics.

	SUV below 10 (n = 21)		SUV 10 to 14 (n = 24)		SUV 15 or above (n = 26)		p-value
	Mean	Sd	Mean	Sd	Mean	Sd	
Age at diagnosis in years	62.6	12.1	61.7	12.8	66.8	10.3	0.26
Sex							
Male	14	66.7%	15	62.5%	11	42.3%	
Female	7	33.3%	9	37.5%	15	57.7%	0.20
Smoking status							
Never	2	9.5%	2	8.3%	2	7.7%	
Current	13	61.9%	9	37.5%	8	30.8%	
Former	5	23.9%	11	45.8%	14	53.8%	
unknown	1	4.7%	2	8.3%	2	7.7%	0.51
PD-L1%							
<1%	10	47.6%	6	25.0%	5	19.2%	
1 to 49%	6	28.6%	11	45.8%	14	53.8%	
> 50%	5	23.8%	7	29.2%	7	26.9%	0.26
Histology							
Adenocarcinoma	18	85.7%	19	73.1%	24	92.3%	0.40
Squamos carcinoma	3	14.3%	7	26.9%	2	7.7%	0.54
Metastases site							
Adrenal	4	19.0%	6	25.0%	9	34.6%	0.40
Bone	11	52.4%	8	33.3%	12	46.2%	0.54
Brain	12	57.1%	8	33.3%	10	38.5%	0.40
Soft tissue	0	0.0%	0	0.0%	1	3.8%	1.00
Liver	1	4.8%	0	0.0%	3	11.5%	0.26
Peritoneum	1	4.8%	0	0.0%	2	7.7%	0.64
Pleura	3	14.3%	5	20.8%	3	11.5%	0.71
Muscle	2	9.5%	1	4.2%	1	3.8%	0.68
Lung	0	0.0%	2	8.3%	1	3.8%	0.52
Renal	4	19.0%	6	25.0%	9	34.6%	0.40
Pericardal	0	0.0%	1	4.2%	0	0.0%	0.64
Spleen	0	0.0%	1	4.2%	0	0.0%	0.64
Lymphnodes	3	14.3%	0	0.0%	3	11.5%	0.20
Number of metastases							
None	0	0.0%	0	0.0%	1	3.8%	1.00
1	13	61.9%	17	70.8%	10	38.5%	0.06
2	4	19.0%	5	20.8%	11	42.3%	0.18
3	3	14.3%	1	4.2%	2	7.7%	0.59
4	0	0.0%	0	0.0%	2	7.7%	0.33
5	1	4.8%	1	4.2%	0	0.0%	0.54
TTF-1							
positive	17	80.9%	19	73.1%	24	92.3%	
negative	4	19.0%	7	26.9%	2	7.7%	0.21
Grading							
Grade 1	0	0.0%	0	0.0%	0	0.0%	
Grade 2	1	4.8%	2	8.3%	2	7.7%	
Grade 3	2	9.5%	6	25.0%	6	23.1%	
unknown	18	85.7%	16	66.7%	18	69.2%	1.00

PD-L1, programmed death-ligand 1; SUV, standardized uptake value; SD, standard deviation; TTF-1, thyroid transcription factor 1.

Smoking history was similarly distributed across SUV categories (p = 0.51). PD-L1 expression levels were also balanced, with no statistically significant differences observed among groups (p = 0.26). Likewise, the distribution of metastatic sites—including adrenal, bone, brain, liver, pleura, lung, and lymph node involvement—did not differ significantly according to SUV category (p > 0.20), indicating no evident association between baseline SUV level and pattern of metastatic spread.

With respect to histology, adenocarcinoma was the predominant subtype, accounting for approximately 85% of cases, followed by squamous cell carcinoma and other histologies. TTF-1 expression and tumor grading were similarly distributed across SUV groups, without significant differences.

Overall, 53 patients received chemo-immunotherapy, whereas 18 patients received immune checkpoint inhibitor monotherapy. Baseline characteristics according to treatment group are summarized in **Supplementary Table 2**. Patients treated with ICI monotherapy were older and showed higher PD-L1 expression, whereas PET-derived metabolic parameters did not differ significantly between treatment groups. Details of the treatment regimens administered are summarized in **Supplementary Table 7**.

### 3.2 Response to treatment according to PET biomarkers

The pattern of treatment responses varied across baseline SUV strata, indicating differing response distributions between groups. Patients with SUV ≥15 exhibited the highest partial response (PR) rate (76.0%), compared with 69.6% in the SUV<10 group and 65.2% in those with SUV 10–14. The highest proportion of progressive disease (PD) as best response was observed in the SUV 10–14 group (30.4%), compared with lower rates in the SUV<10 and SUV ≥15 groups (17.4% and 12.0%, respectively). However, these variations in response patterns were not statistically significant (p > 0.3) (**Table 2**). The proportion of patients developing progressive disease during follow-up also varied numerically across SUV groups, being lowest in the SUV<10 cohort and higher in the two groups with SUV ≥10, but without statistical significance (p=0.39).

**Table 2:** Response to treatment.

	SUV below 10 (n = 23)		SUV 10 to 14 (n = 23)		SUV 15 or above (n = 25)		p-value
	n	%	n	%	n	%	
Best response							
PR	16	69.6%	15	65.2%	19	76.0%	0.76
SD	2	8.7%	1	4.3%	3	12.0%	0.77
PD	4	17.4%	7	30.4%	3	12.0%	0.32
unknown	1	4.3%	0	0.0%	0	0.0%	
Progressive disease							
yes	11	47.8%	15	65.2%	15	60.0%	
no	9	39.1%	3	13.0%	6	24.0%	
unknown	3	13.0%	5	21.7%	4	16.0%	0.39
	ratio SUV below 3.33 (n = 36)		ratio SUV above 3.33 (n = 37)		p-value		
	n	%	n	%			
Best response							
PR	20	55.6%	30	81.1%	0.02	*	
SD	3	8.3%	3	8.1%	1.00		
PD	12	33.3%	2	5.4%	0.01	**	
unknown	0	0.0%	1	2.7%			
Progressive disease							
yes	21	58.3%	20	54.1%			
no	9	25.0%	9	24.3%			
unknown	5	13.9%	7	18.9%	0.90		

PD, progressive disease; PR, partial response; SD, stable disease; SUV, standardized uptake value.

A more pronounced relationship with treatment outcomes was observed for MTV. PR rates decreased with increasing MTV, from 88.9% in patients with MTV ≤110 to 50.0% in those with MTV >606. Moreover, the likelihood of PD as best response increased with MTV, reaching 38.9% in the MTV 291–606 group and 27.8% in the MTV >606 group, with a significant association across MTV categories (p=0.04). Similarly, the proportion of patients experiencing progressive disease during follow-up rose steadily with MTV, from 38.9% in the lowest MTV group to 77.8% in the highest, also reaching statistical significance (p=0.04).

Overall, while higher SUV values were numerically associated with greater response rates, only MTV demonstrated a statistically significant association with disease progression, suggesting that tumor metabolic burden may be more closely associated with disease progression than uptake intensity in this exploratory cohort. Correlation analyses between imaging and systemic inflammatory biomarkers are presented in **Table 3**.

**Table 3:** Correlation between SUV, MTV with LDH, CRP and NLR.

	SUV	
	rho	p-value
LDH	0.08	0.52
CRP	0.43	0.0002
NLR	0.03	0.79
	ratio SUV	
	rho	p-value
LDH	0.10	0.40
CRP	0.12	0.33
NLR	-0.06	0.64
	MVT	
	rho	p-value
LDH	0.40	0.0008
CRP	0.20	0.10
NLR	0.12	0.36

CRP, C-reactive protein; LDH, lactate dehydrogenase; MTV, metabolic tumor volume; NLR, neutrophil-to-lymphocyte ratio; SUV, standardized uptake value.

Distinct associations emerged between PET-derived metrics and circulating biomarkers.

SUVmax showed a moderate positive association with higher baseline serum CRP levels (p = 0.0002), while no relationships were observed with LDH (p = 0.52) or NLR (p = 0.79). The SUV-to-tumor-size ratio was not associated with any of the evaluated biomarkers, including LDH (p = 0.40), CRP (p = 0.33), and NLR (p = 0.64).

In contrast, MTV was significantly associated with LDH levels (p = 0.0008), whereas no significant relationships were observed with higher baseline serum CRP levels (p = 0.10) or NLR (p = 0.36).

**Figure 1:**
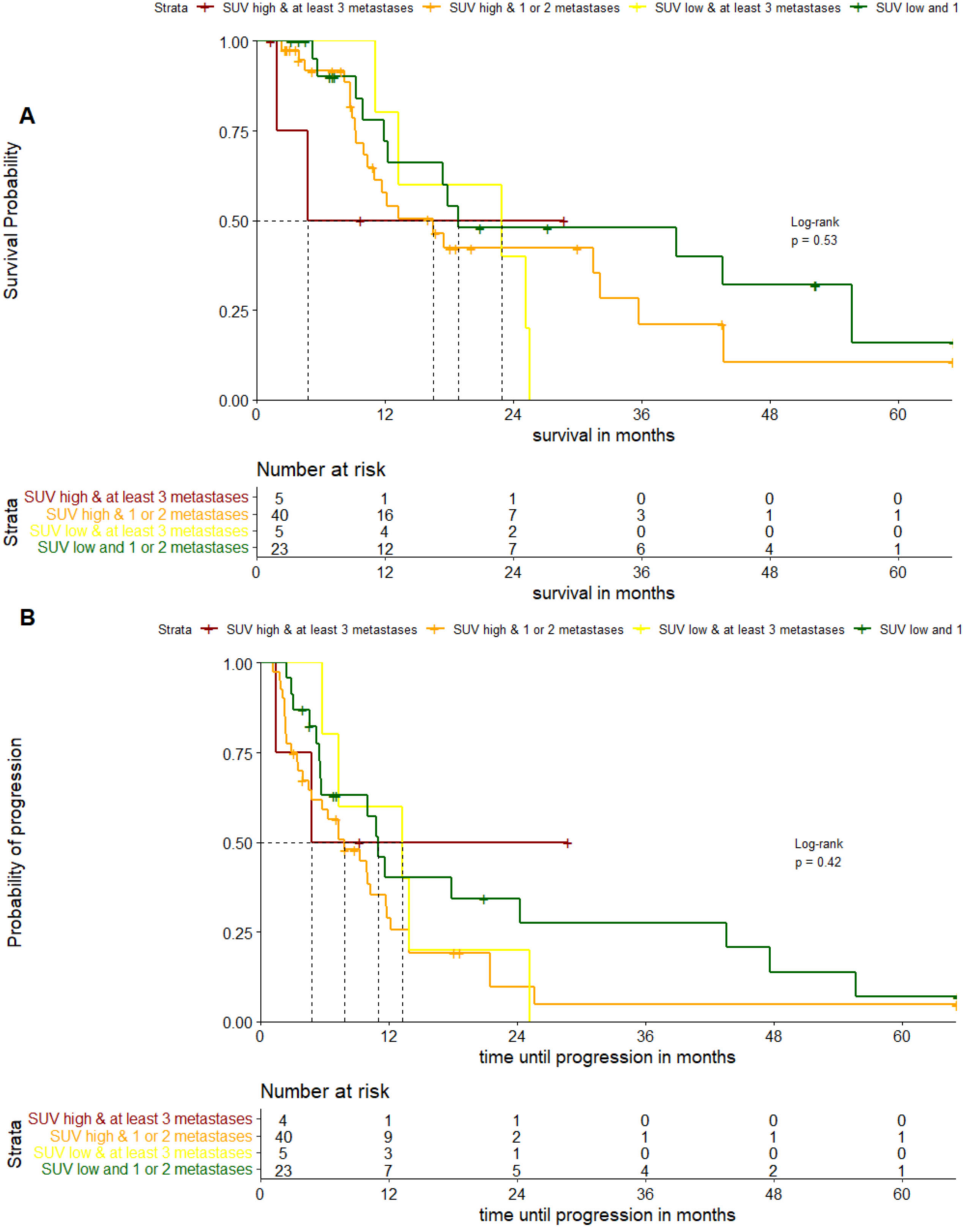
Kaplan-Meier curves of overall and progression free survival according to SUV and number of metastases.

Overall, SUVmax was associated with CRP, while MTV correlated with LDH, whereas no significant associations were observed for NLR, suggesting that metabolic activity and tumor volume may reflect different aspects of tumor biology. These relationships are illustrated in **Figure 1**, which demonstrates overall weak-to-moderate correlations between PET-derived and inflammatory biomarkers.

### 3.3 Survival outcomes according to PET biomarkers and metastatic burden

At the time of analysis, 39 of 71 patients (54.9%) had died and 54 of 71 patients (76.1%) had experienced disease progression or death, corresponding to the events included in the OS and PFS analyses, respectively.

**Figure 2:**
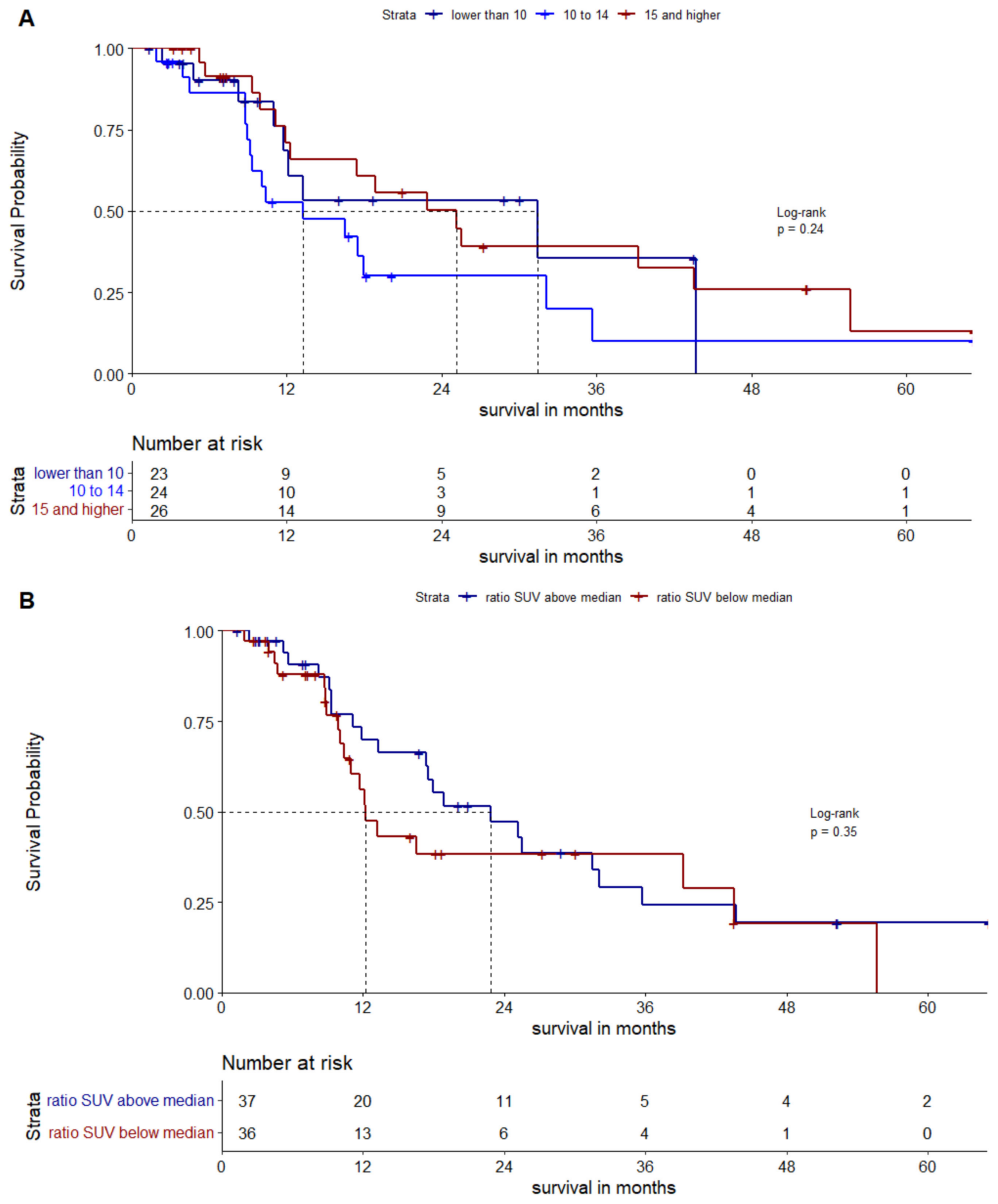
Kaplan-Meier curves of overall survival.

**Figure 3:**
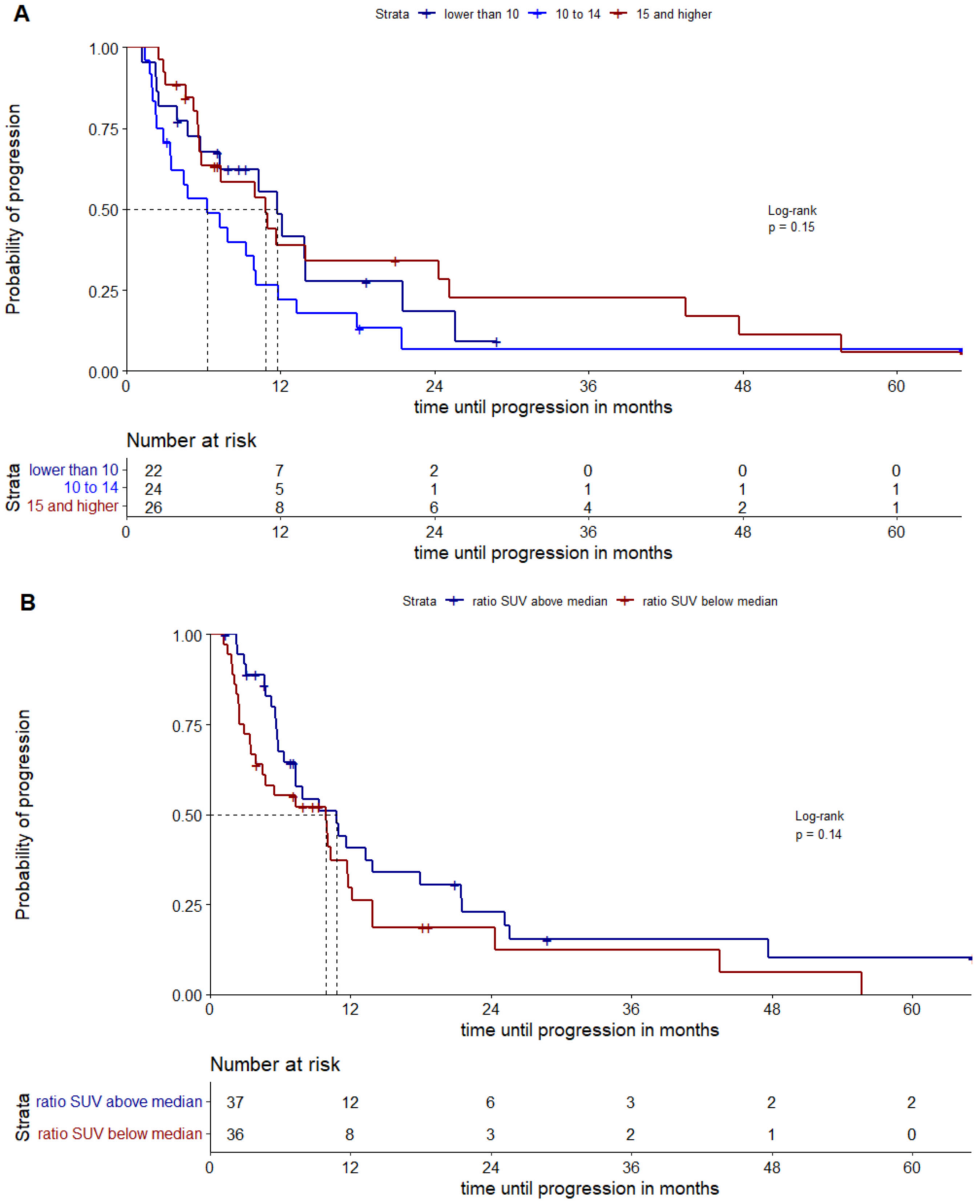
Kaplan-Meier curves of progression free survival.

Kaplan–Meier analyses showed no significant association between baseline SUV categories and OS (**Figure 2A**). Although patients with SUV ≥15 appeared to have numerically longer survival, the curves largely overlapped and the difference was not statistically significant (p = 0.24). Likewise, stratification by SUV ratio did not meaningfully discriminate OS (**Figure 2B**, p = 0.35). Similar findings were observed for PFS, where neither SUV categories nor SUV ratio significantly affected time to progression (**Figures 3A, B**; p = 0.15 and p = 0.14, respectively).

Multivariable Cox regression analyses for OS confirmed these observations (**Table 4**). Baseline SUV did not emerge as a significant prognostic factor (HR 1.00, p = 0.91), and the SUV-to-tumor-size ratio was likewise not associated with survival (HR 1.10, p = 0.40). Volumetric tumor burden showed a trend toward prognostic relevance, although MTV as a continuous variable did not reach statistical significance (p = 0.22). .

**Table 4:** Cox regression of overall survival.

Model 1	HR	Beta	Se	Z-value	P-value
SUV	1,00	0,00	0,03	-0,11	0,91
age	1,03	0,03	0,02	1,15	0,25
female vs. male	0,24	-1,44	0,52	-2,78	0,01
current vs. never smoker	1,39	0,33	1,09	0,31	0,76
former vs. never smoker	0,93	-0,07	1,08	-0,07	0,95
unknown vs. never smoker	0,41	-0,90	1,49	-0,61	0,55
PD-L1 expression in %	0,68	-0,38	0,67	-0,57	0,57
adrenal metasases	4,04	1,40	0,58	2,40	0,02
bone metasases	2,405	0,88	0,50	1,74	0,08
brain metasases	1,60	0,47	0,47	1,00	0,32
hepatic metasases	0,82	-0,20	0,89	-0,22	0,82
pleural metasases	1,32	0,28	0,79	0,35	0,72
lung metasases	8,48	2,14	1,00	2,14	0,03
lymphnodes metasases	1,13	0,12	0,88	0,14	0,89
TTF-1 negative vs. positive	5,05	1,62	0,62	2,60	0,01
Model 2	HR	Beta	Se	Z-value	P-value
ratio SUV/tumor size	1,10	0,10	0,12	0,84	0,40
age	1,03	0,03	0,02	1,30	0,19
female vs. male	0,21	-1,56	0,53	-2,97	0,00
current vs. never smoker	1,63	0,49	1,11	0,44	0,66
former vs. never smoker	1,04	0,04	1,07	0,04	0,97
unknown vs. never smoker	0,39	-0,95	1,47	-0,65	0,52
PD-L1 expression in %	0,52	-0,66	0,72	-0,91	0,36
adrenal metasases	4,24	1,44	0,59	2,45	0,01
bone metasases	2,28	0,826	0,50	1,66	0,10
brain metasases	1,50	0,40	0,47	0,86	0,39
hepatic metasases	0,79	-0,23	0,89	-0,26	0,79
pleural metasases	1,32	0,28	0,79	0,35	0,72
lung metasases	10,27	2,33	1,02	2,29	0,02
lymphnodes metasases	1,31	0,27	0,89	0,30	0,76
TTF-1 negative vs. positive	5,29	1,67	0,62	2,68	0,01
Model 3	HR	Beta	Se	Z-value	P-value
MVT	1,00	0,00	0,00	1,22	0,22
age	1,04	0,04	0,02	1,50	0,13
female vs. male	0,17	-1,78	0,55	-3,27	0,001
current vs. never smoker	0,47	-0,75	1,26	-0,59	0,55
former vs. never smoker	0,33	-1,11	1,28	-0,86	0,39
unknown vs. never smoker	0,12	-2,08	1,63	-1,28	0,20
PD-L1 expression in %	0,57	-0,56	0,73	-0,77	0,44
adrenal metasases	3,86	1,35	0,60	2,25	0,02
bone metasases	1,62	0,48	0,54	0,90	0,37
brain metasases	1,88	0,63	0,52	1,23	0,22
hepatic metasases	1,62	0,48	0,99	0,49	0,63
pleural metasases	0,57	-0,56	0,95	-0,59	0,56
lung metasases	10,09	2,31	1,01	2,29	0,02
lymphnodes metasases	1,40	0,34	0,94	0,36	0,72
TTF-1 negative vs. positive	6,39	1,85	0,70	2,65	0,01

MTV, metabolic tumor volume; PD-L1, programmed death-ligand 1; SUV, standardized uptake value; TTF-1, thyroid transcription factor 1.

**Figure 4:**
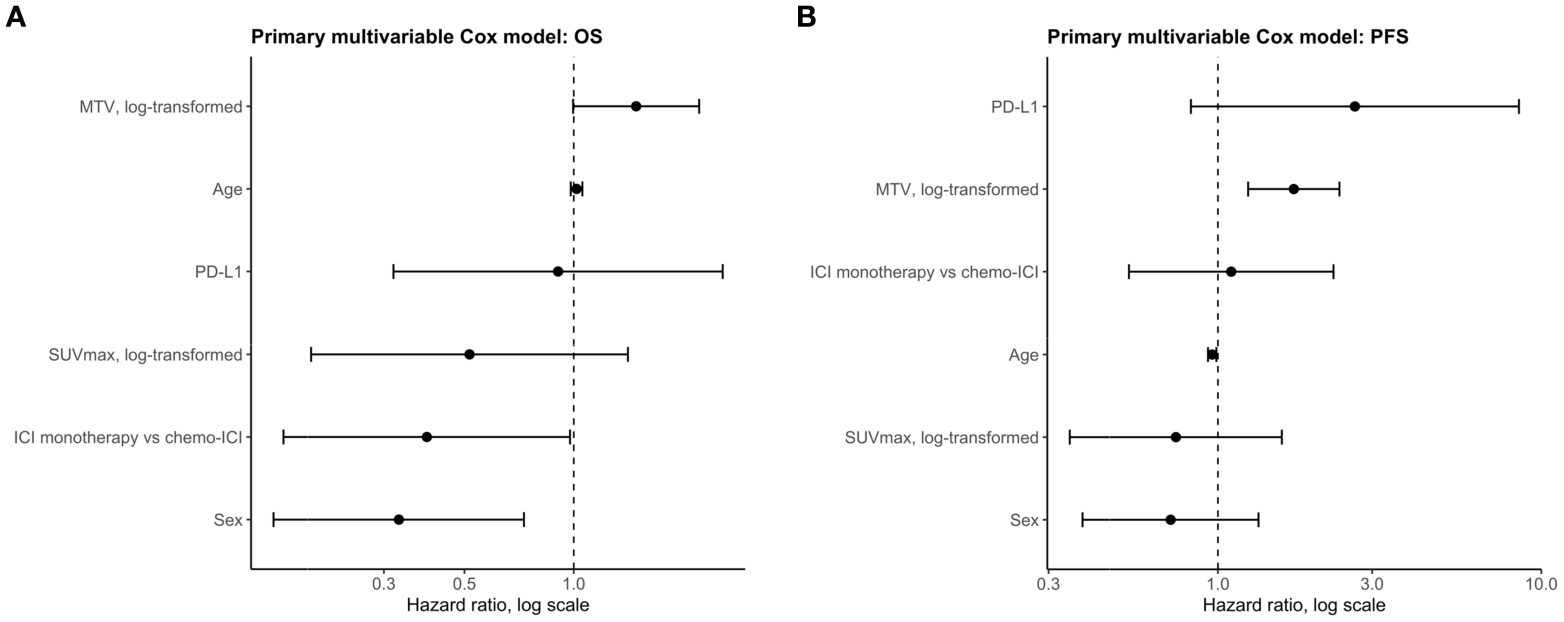
**(A)** Forest plot of the predefined primary multivariable Cox proportional hazards model for overall survival (OS). The model included log-transformed SUVmax, log-transformed MTV, age, sex, PD-L1 expression and treatment group. Error bars indicate 95% confidence intervals and the dashed line indicates HR = 1. **(B)** Forest plot of the predefined primary multivariable Cox proportional hazards model for progression-free survival (PFS). The model included log-transformed SUVmax, log-transformed MTV, age, sex, PD-L1 expression and treatment group. Error bars indicate 95% confidence intervals and the dashed line indicates HR = 1.

The results of the predefined primary multivariable Cox model for overall survival are summarized in **Figure 4A**.

In contrast, we observed that several clinical variables were independently associated with survival. After adjustment for SUVmax, female sex was independently associated with improved OS (HR 0.24, p = 0.01), whereas adrenal metastases (HR 4.04, p = 0.02), lung metastases (HR 8.48, p = 0.03), and absence of TTF-1 expression (HR 5.05, p = 0.01) were associated with significantly worse outcomes.

Comparable findings were observed after adjustment for the SUV-to-tumor-size ratio, female sex remained protective (HR 0.21, p< 0.01) and adrenal metastases (HR 4.24, p = 0.01), lung metastases (HR 10.27, p = 0.02), and TTF-1 negativity (HR 5.29, p = 0.01) continued to predict poorer outcomes.

Similarly, after adjustment for MTV, female sex was associated with improved prognosis (HR 0.17, p = 0.001), whereas the presence of adrenal metastases (HR 3.86, p = 0.02), lung metastases (HR 10.09, p = 0.02), and lack of TTF-1 expression (HR 6.39, p = 0.01) were associated with significantly worse survival.

For PFS, similar patterns were observed (**Table 5**). Neither SUV nor SUV-derived metrics predicted progression risk. After adjustment for SUVmax, the presence of lung metastases (HR 5.07, p = 0.05) and lack of TTF-1 expression (HR 3.25, p = 0.01) were associated with shorter PFS.

**Table 5:** Cox regression of progression-free survival.

Model 1	HR	Beta	Se	Z-value	P-value	
SUV	1,01	0,01	0,02	0,44	0,66	
age	1,00	0,00	0,02	0,08	0,94	
female vs. male	0,62	-0,47	0,41	-1,15	0,25	
current vs. never smoker	1,31	0,27	0,69	0,39	0,70	
former vs. never smoker	0,84	-0,17	0,72	-0,24	0,81	
unknown vs. never smoker	1,05	0,05	0,97	0,05	0,96	
PD-L1 expression in %	0,60	-0,51	0,57	-0,91	0,37	
adrenal metasases	0,92	-0,09	0,46	-0,19	0,85	
bone metasases	1,352	0,30	0,42	0,72	0,47	
brain metasases	1,66	0,51	0,40	1,26	0,21	
hepatic metasases	1,14	0,13	0,73	0,18	0,86	
pleural metasases	2,11	0,75	0,65	1,15	0,25	
lung metasases	5,07	1,62	0,82	1,97	0,05	*
lymphnodes metasases	1,21	0,19	0,76	0,25	0,81	
TTF-1 negative vs. positive	3,25	1,18	0,47	2,49	0,01	*
Model 2	HR	Beta	Se	Z-value	P-value	
ratio SUV/tumor size	1,01	0,01	0,07	0,18	0,86	
age	1,00	0,00	0,02	0,10	0,92	
female vs. male	0,65	-0,44	0,40	-1,09	0,28	
current vs. never smoker	1,37	0,32	0,71	0,45	0,65	
former vs. never smoker	0,92	-0,08	0,72	-0,11	0,91	
unknown vs. never smoker	1,13	0,12	0,97	0,12	0,90	
PD-L1 expression in %	0,61	-0,50	0,58	-0,85	0,40	
adrenal metasases	0,93	-0,08	0,46	-0,17	0,87	
bone metasases	1,378	0,32	0,41	0,78	0,44	
brain metasases	1,67	0,51	0,40	1,30	0,20	
hepatic metasases	1,15	0,14	0,73	0,19	0,85	
pleural metasases	2,23	0,80	0,64	1,25	0,21	
lung metasases	4,93	1,60	0,83	1,93	0,05	.
lymphnodes metasases	1,19	0,17	0,76	0,22	0,82	
TTF-1 negative vs. positive	3,20	1,16	0,47	2,46	0,01	*
Model 3	HR	Beta	Se	Z-value	P-value	
MVT	1,00	0,00	0,00	1,95	0,05	
age	1,01	0,01	0,02	0,66	0,51	
female vs. male	0,50	-0,69	0,42	-1,63	0,10	
current vs. never smoker	0,62	-0,47	0,68	-0,70	0,48	
former vs. never smoker	0,40	-0,91	0,71	-1,28	0,20	
unknown vs. never smoker	0,49	-0,71	0,98	-0,72	0,47	
PD-L1 expression in %	0,40	-0,92	0,64	-1,45	0,15	
adrenal metasases	0,86	-0,16	0,48	-0,32	0,75	
bone metasases	1,02	0,02	0,44	0,05	0,96	
brain metasases	2,01	0,70	0,44	1,60	0,11	
hepatic metasases	2,94	1,08	0,70	1,53	0,13	
pleural metasases	1,38	0,32	0,69	0,46	0,64	
lung metasases	7,51	2,02	0,81	2,48	0,01	*
lymphnodes metasases	1,45	0,37	0,79	0,47	0,64	
TTF-1 negative vs. positive	4,16	1,43	0,50	2,87	0,004	**

MTV, metabolic tumor volume; PD-L1, programmed death-ligand 1; SUV, standardized uptake value; TTF-1, thyroid transcription factor 1.

The corresponding predefined multivariable Cox model for progression-free survival is presented in **Figure 4B**.

Comparable findings were observed after adjustment for the SUV-to-tumor-size ratio, lung metastases (HR 4.93, p = 0.05) and TTF-1 negativity (HR 3.20, p = 0.01) remained associated with worse outcomes.

When MTV was included in the model, higher tumor burden showed a borderline association with shorter PFS (p = 0.05). In this setting, lung metastases (HR 7.51, p = 0.01) and absence of TTF-1 expression (HR 4.16, p = 0.004) were independently associated with inferior PFS.

Overall, PET-derived parameters did not demonstrate a consistent independent association with PFS, whereas lung metastatic involvement and TTF-1 status emerged as the most robust clinical predictors of progression.

When survival outcomes were explored according to the combined effect of SUV level and metastatic burden (**Figure 1**), no statistically significant differences were observed for either OS or PFS (p = 0.53 and p = 0.42, respectively). Nevertheless, descriptive inspection suggested that patients with lower SUV values and limited metastatic spread tended to experience more favorable long-term outcomes, whereas those with higher SUV and multiple metastatic sites showed earlier progression and death.

### 3.4 Integrated prognostic modeling and model performance

To further investigate the joint prognostic value of metabolic PET biomarkers and systemic inflammatory markers, additional multivariable Cox regression analyses were performed incorporating PET-derived variables (SUVmax and MTV), inflammatory biomarkers (CRP, LDH and NLR), and clinical covariates within the same analytical framework. These analyses were performed as secondary exploratory analyses to complement the category-based models presented above. Continuous log-transformed variables were used for SUVmax, MTV, CRP, LDH, and NLR in order to preserve statistical information and avoid potential bias related to arbitrary categorization. Categorized PET variables were used exclusively for descriptive visualization and exploratory analyses, whereas all primary prognostic models were based on continuous log-transformed biomarker values. Missing data were limited across all major study variables, with missingness rates of 4.2% for MTV, 4.2% for NLR, 2.8% for PD-L1 expression, and 1.4% for both CRP and LDH. A detailed overview of missingness across all study variables is provided in **Supplementary Table 5**. Given the low proportion of missing values, complete-case analyses were performed.

Model discrimination was assessed using Harrell’s concordance index (C-index), together with Akaike Information Criterion (AIC) and Bayesian Information Criterion (BIC) (**Table 6**). For overall survival, the combined model including clinical variables, PET biomarkers, and inflammatory markers demonstrated the highest discriminative performance (C-index 0.745), compared with the clinical-only model (C-index 0.697), the PET-only model (C-index 0.612), and the inflammation-only model (C-index 0.651). Similar findings were observed for progression-free survival, where the combined model achieved the highest predictive accuracy (C-index 0.750), compared with the clinical-only (0.655), PET-only (0.630), and inflammation-only (0.633) models. Despite the improved performance of the integrated models, none of the individual PET-derived or inflammatory biomarkers remained independently associated with OS or PFS after full adjustment, suggesting that these biomarkers may capture partially overlapping biological information rather than providing strong independent prognostic effects.

**Table 6:** Comparison of prognostic model performance for OS and PFS.

Outcome	Model	n	Events	Predictors_df	EPV	AIC	BIC	C_index
OS	Clinical base	68	37	8	4.62	242.2197	255.107	0.69685
OS	PET only	68	38	2	19	245.042	248.3171	0.612263
OS	Inflammation only	68	36	3	12	234.4545	239.2051	0.65131
OS	Combined	62	33	13	2.54	212.6227	232.0773	0.745355
OS	Spline combined	62	33	17	1.94	208.4413	233.8819	0.769399
PFS	Clinical base	67	51	8	6.38	363.2418	378.6964	0.654995
PFS	PET only	67	53	2	26.5	365.3057	369.2462	0.62963
PFS	Inflammation only	67	51	3	17	355.999	361.7945	0.633203
PFS	Combined	61	47	13	3.62	310.772	334.8239	0.749706
PFS	Spline combined	61	47	17	2.76	312.1561	343.6087	0.771496

AIC, Akaike Information Criterion; BIC, Bayesian Information Criterion; C-Index, Harrell’s concordance index; EPV, events per variable; OS, overall survival; PET, positron emission tomography, PFS, progression-free survival; SE, standard error.

Correlations between PET-derived biomarkers and inflammatory markers are shown in Supplementary **Figure 1**. Overall, only weak to moderate correlations were observed, indicating that these biomarkers provide complementary rather than redundant information.

**Figure 5:**
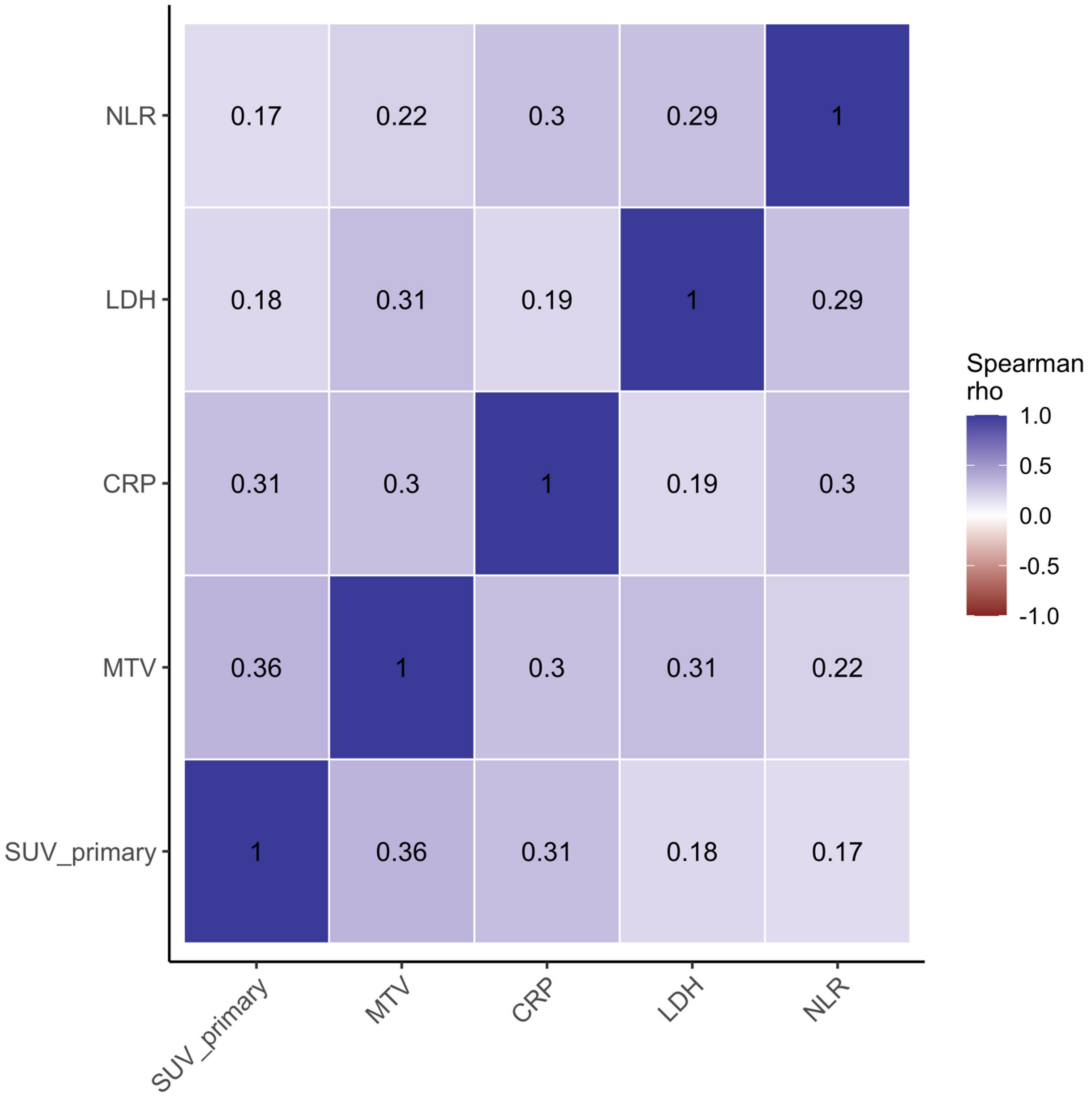
Spearman correlation heatmap of baseline PET-derived parameters (SUVprimary and MTV) and systemic inflammatory biomarkers (CRP, LDH and NLR).

To explore potential nonlinear relationships between biomarkers and survival outcomes, restricted cubic spline analyses were performed. For overall survival, spline modeling significantly improved model fit compared with linear modeling (likelihood ratio test p = 0.016; FDR-adjusted p = 0.032), indicating the presence of potential nonlinear associations between biomarker values and mortality risk. The correlation matrix is presented as a heatmap in **Figure 5**. In contrast, no significant improvement was observed for progression-free survival, suggesting that linear modeling adequately described the relationship between biomarker values and progression risk in the present cohort (**Table 7**).

**Table 7:** Likelihood ratio test comparison of nested prognostic models for overall survival and progression-free survival.

Outcome	Comparison	n_reduced	n_full	loglik_reduced	loglik_full	Chisq	df	p.value
OS	Clinical base vs combined	62	62	-96.1119	-93.3113	5.601194	5	0.346977
OS	Linear combined vs spline combined	62	62	-93.3113	-87.2088	12.20499	4	0.01589
PFS	Clinical base vs combined	61	61	-153.497	-142.386	22.22121	5	0.000475
PFS	Linear combined vs spline combined	61	61	-142.386	-139.077	6.618789	4	0.157458

Chisq, chi-square statistic; df, degrees of freedom; FDR_p, p-value adjusted for multiple testing using the Benjamini–Hochberg method; loglik, log-likelihood; n_full, number of patients in the full model; n_reduced, number of patients in the reduced model; OS, overall survival; PFS, progression-free survival.

Exploratory interaction analyses were subsequently conducted to evaluate whether the prognostic effects of PET biomarkers varied according to systemic inflammatory status or tumor immune characteristics. No significant interactions were observed between PET biomarkers and inflammatory markers (CRP, LDH, or NLR) after adjustment for multiple testing. However, significant interactions were identified between PET biomarkers and PD-L1 expression for overall survival. Both the SUVmax × PD-L1 interaction and the MTV × PD-L1 interaction remained significant after false discovery rate correction (FDR-adjusted p = 0.026 for both comparisons), suggesting that the prognostic relevance of tumor metabolic activity may depend on the underlying immune phenotype of the tumor (**Supplementary Table 2**).

The proportional hazards assumption was formally evaluated using Schoenfeld residual testing and no significant violations were detected. Global tests were non-significant for both OS (p = 0.311) and PFS (p = 0.462), supporting the appropriateness of Cox proportional hazards modeling in the present dataset (**Supplementary Table 3**). To further assess model stability and the potential impact of overfitting, events-per-variable ratios were calculated and penalized Cox regression using LASSO regularization was performed. The combined models yielded events-per-variable ratios below conventional recommendations, suggesting an increased risk of model overfitting and reduced coefficient stability. Consistent with this observation, LASSO regression did not retain any variables at the λ1se threshold for either OS or PFS, indicating that the identified prognostic associations may be sensitive to model penalization and should therefore be interpreted cautiously (**Supplementary Table 4**).

To further assess internal model stability, bootstrap resampling was performed using 300 iterations. The combined models demonstrated mean bootstrap C-index values of 0.814 for OS and 0.793 for PFS, with all bootstrap iterations converging successfully, supporting the overall robustness of model discrimination despite the limited sample size (**Supplementary Table 6**).

Overall, these analyses indicate that the integration of clinical characteristics, PET-derived metabolic parameters, and systemic inflammatory biomarkers improves prognostic discrimination compared with individual biomarker domains alone. Nevertheless, the absence of independently significant PET or inflammatory biomarkers in fully adjusted models suggests substantial biological overlap among these variables and supports a cautious interpretation of their prognostic role.

## 4 Discussion

In this retrospective study, we evaluated the prognostic and predictive value of baseline ^18F-FDG PET/CT-derived metabolic parameters together with systemic inflammatory biomarkers in patients with advanced NSCLC receiving first-line ICIs. Our findings indicate that conventional PET biomarkers and inflammatory markers provide complementary information regarding tumor burden, host inflammatory status, and treatment response, but do not independently outperform established clinical prognostic factors after comprehensive multivariable adjustment. Importantly, integrated models combining clinical, metabolic, and inflammatory variables demonstrated better prognostic discrimination than models based on individual biomarker domains. Exploratory analyses restricted to the MTV cohort yielded qualitatively similar findings, with MTV remaining associated with both OS and PFS in adjusted models (**Supplementary Table 8**). Exploratory interaction analyses evaluated potential effect modification by PD-L1 expression (**Supplementary Table 9**). Given the limited sample size and multiple testing burden, these findings should be considered hypothesis-generating. Restricted cubic spline analyses did not demonstrate convincing evidence of clinically relevant non-linear associations between PET biomarkers and survival outcomes, supporting the use of continuous log-transformed PET variables in the primary models (**Supplementary Table 10**).

Tumor glucose metabolism quantified by SUVmax reflects not only tumor cell proliferation but also the metabolic activity of the tumor microenvironment, including infiltrating immune and stromal cells. Historically, elevated FDG uptake has been considered a marker of aggressive tumor biology and adverse prognosis. However, increasing evidence from the immunotherapy era suggests that FDG uptake may also reflect immune activation and inflammatory signaling within the tumor microenvironment. Activated T lymphocytes and myeloid cells exhibit high glycolytic activity, and therefore elevated FDG uptake may partly represent ongoing antitumor immune responses rather than tumor aggressiveness alone (17–19). Recent studies have further demonstrated that PET imaging may capture dynamic tumor–immune interactions during immune checkpoint blockade and may contribute to the identification of atypical response patterns such as pseudoprogression and dissociated responses (20, 21).

In our cohort, higher SUV values were associated with numerically higher objective response rates but did not translate into improved survival outcomes. This observation is consistent with the hypothesis that metabolically active tumors may exhibit increased immunogenicity and therefore greater susceptibility to immune checkpoint blockade, although these potential benefits may be counterbalanced by adverse biological features associated with aggressive disease (22). The absence of an independent prognostic association after multivariable adjustment suggests that SUVmax alone is insufficient to capture the complexity of tumor–host interactions determining long-term outcomes during immunotherapy.

Unlike SUV intensity measurements, MTV provides a quantitative estimate of global tumor burden. In the present study, increasing MTV was associated with higher rates of progressive disease and earlier progression, supporting the concept that tumor burden remains a major determinant of immunotherapy efficacy. Large tumor burden may contribute to immune exhaustion, T-cell dysfunction and the development of an immunosuppressive tumor microenvironment, thereby limiting the effectiveness of immune checkpoint inhibition (23, 24). Previous studies have consistently demonstrated associations between elevated MTV and inferior outcomes in NSCLC patients treated with ICIs (15, 24, 25). More recently, attention has shifted toward whole-body metabolic burden metrics, including total lesion glycolysis and disseminated metabolic tumor volume, which may better reflect the systemic extent of disease and tumor–host interactions than focal measurements alone (26, 27). Nevertheless, MTV did not retain independent prognostic significance in our fully adjusted models, suggesting that the prognostic information conveyed by tumor burden substantially overlaps with established clinical characteristics and systemic inflammatory markers.

An additional observation of our study was the relationship between tumor metabolism and systemic inflammation. We observed significant correlations between SUVmax and CRP levels, supporting the hypothesis that tumor metabolic activity and inflammatory signaling represent closely interconnected biological processes. Systemic inflammatory biomarkers such as CRP, NLR and LDH have been associated with tumor burden, cytokine activation and immune suppression in NSCLC (28–32). Furthermore, experimental studies suggest that metabolic competition between tumor cells and immune effector cells within the tumor microenvironment may directly influence sensitivity to immune checkpoint blockade (18, 19). However, none of the inflammatory biomarkers retained independent prognostic significance after comprehensive adjustment, indicating that these markers likely capture overlapping aspects of tumor biology rather than distinct prognostic pathways.

One of the most relevant findings of the present study was the superior performance of integrated prognostic models combining clinical characteristics, PET-derived metrics and inflammatory biomarkers. Combined models consistently outperformed PET-only and inflammation-only approaches for both overall survival and progression-free survival. These findings suggest that metabolic imaging and inflammatory biomarkers should not be considered standalone prognostic tools but rather complementary sources of biological information that may contribute additional context when interpreted alongside established clinical factors.

One of the most biologically intriguing findings of the present study was the observed interaction between PET-derived metabolic biomarkers and PD-L1 expression, particularly for MTV in relation to overall survival. Although exploratory and requiring external validation, these findings suggest that the prognostic implications of tumor metabolic burden may differ according to the underlying tumor immune phenotype.

This observation is biologically plausible and consistent with the growing understanding of metabolic reprogramming within the tumor microenvironment. Cancer cells frequently exhibit increased aerobic glycolysis, commonly referred to as the Warburg effect, resulting in increased glucose consumption and FDG uptake even under normoxic conditions (33, 34). Beyond supporting tumor proliferation, metabolic reprogramming also profoundly influences immune regulation and antitumor responses.

Hypoxia-inducible factor 1-alpha (HIF-1α) plays a central role in this process by promoting glycolytic metabolism while simultaneously enhancing PD-L1 expression on tumor cells and immune cells within the tumor microenvironment (35, 36). Experimental evidence suggests that HIF-1α-mediated signaling may directly link tumor hypoxia, glucose metabolism and immune escape mechanisms, thereby providing a biological explanation for the interaction observed between FDG uptake and tumor immune phenotype.

Furthermore, tumor glycolysis may influence both the composition and function of infiltrating immune cells. Highly glycolytic tumors compete with activated T lymphocytes for glucose availability within the tumor microenvironment, while lactate accumulation promotes T-cell dysfunction, immune exhaustion and the recruitment of immunosuppressive cell populations (37, 38). Conversely, FDG uptake may also partly reflect the presence of metabolically active immune and inflammatory cells, including activated T lymphocytes and macrophages, highlighting the complexity of interpreting PET-derived metabolic activity in patients receiving immunotherapy (17–19).

These mechanisms may help explain the observed interaction between PD-L1 expression and PET-derived metabolic biomarkers in our cohort. If confirmed in larger prospective studies, such findings would suggest that tumor metabolism and immune activation should not be considered independent biological domains but rather complementary and interconnected determinants of response to ICIs. This concept further supports integrated biomarker strategies combining metabolic imaging and immune profiling for patient stratification and treatment individualization in advanced NSCLC.

Interestingly, significant interactions were observed between PET biomarkers and PD-L1 expression for overall survival. Although these findings should be interpreted cautiously given the relatively limited sample size, they are biologically plausible because glucose metabolism, hypoxia signaling and PD-L1 expression are closely interconnected within the tumor microenvironment. Emerging radiogenomic studies have demonstrated that PET-derived radiomic signatures may predict PD-L1 expression, tumor immune phenotype and responsiveness to ICIs with greater accuracy than conventional PET parameters alone (17, 39–41). These observations suggest that advanced imaging biomarkers may better capture tumor heterogeneity and immune biology than traditional SUV- or MTV-based measurements.

Recent advances in PET radiomics have further expanded the potential role of metabolic imaging in immunotherapy-treated NSCLC. Radiomic approaches can extract quantitative information related to intratumoral heterogeneity, texture and spatial metabolic distribution that is not captured by conventional metabolic indices. Several recent studies have reported encouraging results regarding the prediction of PD-L1 expression, treatment response and survival using PET-based radiomic models (17, 39–52). Similarly, longitudinal PET assessment during treatment may provide dynamic information regarding early response and resistance mechanisms, potentially improving patient stratification and treatment adaptation strategies.

Our findings support a shift from individual biomarkers toward multimodal prognostic strategies integrating clinical characteristics, metabolic imaging, systemic inflammation and molecular profiling. Such approaches are likely to better capture the multidimensional biological determinants of immunotherapy response than any individual biomarker considered in isolation.

An important observation of the present study was the absence of independently significant PET-derived or inflammatory biomarkers after comprehensive multivariable adjustment. Several factors may account for these findings. First, the relatively limited sample size and number of events, particularly within the MTV cohort, may have reduced the statistical power to detect modest independent effects. Second, treatment heterogeneity, including the use of both ICI monotherapy and chemo-immunotherapy regimens, may have attenuated biomarker-specific associations by introducing variability in treatment response mechanisms. Although treatment type was included as an adjustment variable in all primary multivariable analyses, stratified analyses according to treatment regimen should be considered exploratory because of the limited size of treatment-specific subgroups.

Third, quantitative PET analyses were restricted to patients whose baseline imaging was performed at our institution to ensure methodological consistency for volumetric analyses. Although this approach improved internal validity, it reduced the effective sample size and may have introduced selection bias. Only a small number of patients lacked MTV measurements, limiting the interpretability of formal comparisons.

Methodological variability in PET acquisition and quantification may also contribute to inconsistent findings across studies. PET-derived parameters are influenced by acquisition protocols, image reconstruction methods, scanner characteristics and segmentation approaches, particularly for volumetric measurements such as MTV and TLG. Although restricting volumetric analyses to patients scanned at our institution improved methodological consistency, PET standardization remains a major challenge for the clinical implementation of imaging biomarkers.

In addition, total lesion glycolysis (TLG) could not be assessed because neither TLG nor SUVmean was available in the retrospectively collected dataset, precluding reliable reconstruction of this parameter.

These findings suggest that the absence of independent prognostic significance reflects the multifactorial nature of immunotherapy outcomes rather than a lack of biological relevance of these biomarkers.

Several limitations should be acknowledged. First, the retrospective single-centre design introduces the possibility of selection bias and limits generalisability. Second, the relatively small sample size restricted statistical power, particularly for subgroup analyses, interaction testing and multivariable modeling. Moreover, the events-per-variable ratio was below conventional recommendations, raising concerns regarding model stability. Consistent with this, penalized Cox regression using LASSO regularization did not retain any variables at the λ1se threshold, suggesting that the observed prognostic associations should be interpreted cautiously.

Third, quantitative PET analyses were restricted to patients whose baseline imaging was performed at our institution to ensure methodological consistency for volumetric analyses. Although this approach improved internal validity, it reduced the effective sample size and may have introduced selection bias. Fourth, our analyses focused exclusively on baseline PET parameters and did not incorporate whole-body metabolic tumor burden, TLG, radiomic features, spatial metabolic heterogeneity or longitudinal treatment-related metabolic changes. Emerging evidence suggests that these advanced PET-derived biomarkers may provide incremental prognostic information beyond conventional SUV and MTV measurements in patients receiving ICIs (20, 21, 26, 27, 39–42, 53).

Finally, no independent external validation cohort was available to confirm the robustness and generalisability of the observed findings. Consequently, the findings should be considered exploratory and hypothesis-generating. Decision-curve analysis was not performed because clinical utility cannot be reliably inferred from a retrospective single-centre cohort without external validation. Accordingly, the proposed models should not yet be considered suitable for clinical decision-making or implementation in routine practice. Further prospective studies with independent external validation are warranted before clinical application can be considered.

## 5 Conclusion

Baseline PET-derived metabolic parameters and systemic inflammatory biomarkers provide complementary prognostic information regarding tumor burden, host inflammatory status, and treatment response in patients with advanced NSCLC receiving first-line ICIs. However, neither PET-derived nor inflammatory biomarkers independently outperformed established clinical prognostic factors after comprehensive multivariable adjustment. Integrated models incorporating clinical, metabolic, and inflammatory variables showed better prognostic discrimination than clinical variables alone, supporting the value of multimodal risk stratification. Exploratory findings regarding interactions between PD-L1 expression and PET-derived metabolic biomarkers, particularly MTV, further support the potential value of integrated approaches combining metabolic imaging and immune profiling. Overall, these findings support the development of multimodal prognostic strategies integrating clinical, imaging, inflammatory and immune information for patients treated with immunotherapy. Prospective validation in larger independent cohorts is required to determine the incremental value and clinical applicability of such approaches. The proposed models remain exploratory and hypothesis-generating and should not yet be considered suitable for clinical implementation.

## Data Availability

The original contributions presented in the study are included in the article/**Supplementary Material**. Further inquiries can be directed to the corresponding author.
