# Supplementary material for: Baseline 18F-FDG PET/CT metabolic parameters and systemic inflammatory markers in advanced NSCLC treated with first-line immunotherapy: an exploratory retrospective analysis
Source: Front Oncol. 2026 Aug 5;16:1837219. doi: 10.3389/fonc.2026.1837219 (PMC13485752; doi:10.3389/fonc.2026.1837219)
Supplement: Supplementary file 1 [file Table1.docx]

Table 1 Patient characteristics

|  | SUV below 10 (n = 21) | | SUV 10 to 14 (n = 24) | | SUV 15 or above (n = 26) | | p-value |
| --- | --- | --- | --- | --- | --- | --- | --- |
|  | mean | sd | mean | Sd | mean | sd |  |
| **age at diagnosis in years** | 62.6 | 12.1 | 61.7 | 12.8 | 66.8 | 10.3 | 0.26 |
| **Sex** |  |  |  |  |  |  |  |
| Male | 14 | 66.7% | 15 | 62.5% | 11 | 42.3% |  |
| Female | 7 | 33.3% | 9 | 37.5% | 15 | 57.7% | 0.20 |
| **smoking status** |  |  |  |  |  |  |  |
| Never | 2 | 9.5% | 2 | 8.3% | 2 | 7.7% |  |
| Current | 13 | 61.9% | 9 | 37.5% | 8 | 30.8% |  |
| Former | 5 | 23.9% | 11 | 45.8% | 14 | 53.8% |  |
| unknown | 1 | 4.7% | 2 | 8.3% | 2 | 7.7% | 0.51 |
| **PD-L1 %** |  |  |  |  |  |  |  |
| <1% | 10 | 47.6% | 6 | 25.0% | 5 | 19.2% |  |
| 1 to 49% | 6 | 28.6% | 11 | 45.8% | 14 | 53.8% |  |
| > 50% | 5 | 23.8% | 7 | 29.2% | 7 | 26.9% | 0.26 |
| **histology**  Adenocarcinoma | 18 | 85.7% | 19 | 73.1% | 24 | 92.3% | 0.40 |
| Squamos carcinoma | 3 | 14.3% | 7 | 26.9% | 2 | 7.7% | 0.54 |
| **metastases site** |  |  |  |  |  |  |  |
| Adrenal | 4 | 19.0% | 6 | 25.0% | 9 | 34.6% | 0.40 |
| Bone | 11 | 52.4% | 8 | 33.3% | 12 | 46.2% | 0.54 |
| Brain | 12 | 57.1% | 8 | 33.3% | 10 | 38.5% | 0.40 |
| Soft tissue | 0 | 0.0% | 0 | 0.0% | 1 | 3.8% | 1.00 |
| Liver | 1 | 4.8% | 0 | 0.0% | 3 | 11.5% | 0.26 |
| Peritoneum | 1 | 4.8% | 0 | 0.0% | 2 | 7.7% | 0.64 |
| Pleura | 3 | 14.3% | 5 | 20.8% | 3 | 11.5% | 0.71 |
| Muscle | 2 | 9.5% | 1 | 4.2% | 1 | 3.8% | 0.68 |
| Lung | 0 | 0.0% | 2 | 8.3% | 1 | 3.8% | 0.52 |
| Renal | 4 | 19.0% | 6 | 25.0% | 9 | 34.6% | 0.40 |
| Pericardal | 0 | 0.0% | 1 | 4.2% | 0 | 0.0% | 0.64 |
| Spleen | 0 | 0.0% | 1 | 4.2% | 0 | 0.0% | 0.64 |
| Lymphnodes | 3 | 14.3% | 0 | 0.0% | 3 | 11.5% | 0.20 |
| **number of metastases** |  |  |  |  |  |  |  |
| None | 0 | 0.0% | 0 | 0.0% | 1 | 3.8% | 1.00 |
| 1 | 13 | 61.9% | 17 | 70.8% | 10 | 38.5% | 0.06 |
| 2 | 4 | 19.0% | 5 | 20.8% | 11 | 42.3% | 0.18 |
| 3 | 3 | 14.3% | 1 | 4.2% | 2 | 7.7% | 0.59 |
| 4 | 0 | 0.0% | 0 | 0.0% | 2 | 7.7% | 0.33 |
| 5 | 1 | 4.8% | 1 | 4.2% | 0 | 0.0% | 0.54 |
| **TTF-1** |  |  |  |  |  |  |  |
| positive | 17 | 80.9% | 19 | 73.1% | 24 | 92.3% |  |
| negative | 4 | 19.0% | 7 | 26.9% | 2 | 7.7% | 0.21 |
| **Grading** |  |  |  |  |  |  |  |
| Grade 1 | 0 | 0.0% | 0 | 0.0% | 0 | 0.0% |  |
| Grade 2 | 1 | 4.8% | 2 | 8.3% | 2 | 7.7% |  |
| Grade 3 | 2 | 9.5% | 6 | 25.0% | 6 | 23.1% |  |
| unknown | 18 | 85.7% | 16 | 66.7% | 18 | 69.2% | 1.00 |

Abbreviations: PD-L1, programmed death-ligand 1; SUV, standardized uptake value; SD, standard deviation; TTF-1, thyroid transcription factor 1.

Table 2 Response to treatment

Abbreviations: PD, progressive disease; PR, partial response; SD, stable disease; SUV, standardized uptake value.

Table 3. Correlation between SUV, MTV with LDH, CRP and NLR

**Abbreviations:** CRP, C-reactive protein; LDH, lactate dehydrogenase; MTV, metabolic tumor volume; NLR, neutrophil-to-lymphocyte ratio; SUV, standardized uptake value.

Table 4 Cox regression of overall survival

| model 1 | HR | beta | se | z-value | p-value |  |
| --- | --- | --- | --- | --- | --- | --- |
| SUV | 1,00 | 0,00 | 0,03 | -0,11 | 0,91 |  |
| age | 1,03 | 0,03 | 0,02 | 1,15 | 0,25 |  |
| female vs. male | 0,24 | -1,44 | 0,52 | -2,78 | 0,01 | ** |
| current vs. never smoker | 1,39 | 0,33 | 1,09 | 0,31 | 0,76 |  |
| former vs. never smoker | 0,93 | -0,07 | 1,08 | -0,07 | 0,95 |  |
| unknown vs. never smoker | 0,41 | -0,90 | 1,49 | -0,61 | 0,55 |  |
| PD-L1 expression in % | 0,68 | -0,38 | 0,67 | -0,57 | 0,57 |  |
| adrenal metasases | 4,04 | 1,40 | 0,58 | 2,40 | 0,02 | * |
| bone metasases | 2,405 | 0,88 | 0,50 | 1,74 | 0,08 | . |
| brain metasases | 1,60 | 0,47 | 0,47 | 1,00 | 0,32 |  |
| hepatic metasases | 0,82 | -0,20 | 0,89 | -0,22 | 0,82 |  |
| pleural metasases | 1,32 | 0,28 | 0,79 | 0,35 | 0,72 |  |
| lung metasases | 8,48 | 2,14 | 1,00 | 2,14 | 0,03 | * |
| lymphnodes metasases | 1,13 | 0,12 | 0,88 | 0,14 | 0,89 |  |
| TTF-1 negative vs. positive | 5,05 | 1,62 | 0,62 | 2,60 | 0,01 | ** |
| model 2 | HR | beta | se | z-value | p-value |  |
| ratio SUV/tumor size | 1,10 | 0,10 | 0,12 | 0,84 | 0,40 |  |
| age | 1,03 | 0,03 | 0,02 | 1,30 | 0,19 |  |
| female vs. male | 0,21 | -1,56 | 0,53 | -2,97 | 0,00 | ** |
| current vs. never smoker | 1,63 | 0,49 | 1,11 | 0,44 | 0,66 |  |
| former vs. never smoker | 1,04 | 0,04 | 1,07 | 0,04 | 0,97 |  |
| unknown vs. never smoker | 0,39 | -0,95 | 1,47 | -0,65 | 0,52 |  |
| PD-L1 expression in % | 0,52 | -0,66 | 0,72 | -0,91 | 0,36 |  |
| adrenal metasases | 4,24 | 1,44 | 0,59 | 2,45 | 0,01 | * |
| bone metasases | 2,28 | 0,826 | 0,50 | 1,66 | 0,10 | . |
| brain metasases | 1,50 | 0,40 | 0,47 | 0,86 | 0,39 |  |
| hepatic metasases | 0,79 | -0,23 | 0,89 | -0,26 | 0,79 |  |
| pleural metasases | 1,32 | 0,28 | 0,79 | 0,35 | 0,72 |  |
| lung metasases | 10,27 | 2,33 | 1,02 | 2,29 | 0,02 | * |
| lymphnodes metasases | 1,31 | 0,27 | 0,89 | 0,30 | 0,76 |  |
| TTF-1 negative vs. positive | 5,29 | 1,67 | 0,62 | 2,68 | 0,01 | ** |
| model 3 | HR | beta | se | z-value | p-value |  |
| MVT | 1,00 | 0,00 | 0,00 | 1,22 | 0,22 |  |
| age | 1,04 | 0,04 | 0,02 | 1,50 | 0,13 |  |
| female vs. male | 0,17 | -1,78 | 0,55 | -3,27 | 0,001 | ** |
| current vs. never smoker | 0,47 | -0,75 | 1,26 | -0,59 | 0,55 |  |
| former vs. never smoker | 0,33 | -1,11 | 1,28 | -0,86 | 0,39 |  |
| unknown vs. never smoker | 0,12 | -2,08 | 1,63 | -1,28 | 0,20 |  |
| PD-L1 expression in % | 0,57 | -0,56 | 0,73 | -0,77 | 0,44 |  |
| adrenal metasases | 3,86 | 1,35 | 0,60 | 2,25 | 0,02 | * |
| bone metasases | 1,62 | 0,48 | 0,54 | 0,90 | 0,37 |  |
| brain metasases | 1,88 | 0,63 | 0,52 | 1,23 | 0,22 |  |
| hepatic metasases | 1,62 | 0,48 | 0,99 | 0,49 | 0,63 |  |
| pleural metasases | 0,57 | -0,56 | 0,95 | -0,59 | 0,56 |  |
| lung metasases | 10,09 | 2,31 | 1,01 | 2,29 | 0,02 | * |
| lymphnodes metasases | 1,40 | 0,34 | 0,94 | 0,36 | 0,72 |  |
| TTF-1 negative vs. positive | 6,39 | 1,85 | 0,70 | 2,65 | 0,01 | ** |

Abbreviations: MTV, metabolic tumor volume; PD-L1, programmed death-ligand 1; SUV, standardized uptake value; TTF-1, thyroid transcription factor 1.

Table 5 Cox regression of progression-free survival

| model 1 | HR | beta | se | z-value | p-value |  |
| --- | --- | --- | --- | --- | --- | --- |
| SUV | 1,01 | 0,01 | 0,02 | 0,44 | 0,66 |  |
| age | 1,00 | 0,00 | 0,02 | 0,08 | 0,94 |  |
| female vs. male | 0,62 | -0,47 | 0,41 | -1,15 | 0,25 |  |
| current vs. never smoker | 1,31 | 0,27 | 0,69 | 0,39 | 0,70 |  |
| former vs. never smoker | 0,84 | -0,17 | 0,72 | -0,24 | 0,81 |  |
| unknown vs. never smoker | 1,05 | 0,05 | 0,97 | 0,05 | 0,96 |  |
| PD-L1 expression in % | 0,60 | -0,51 | 0,57 | -0,91 | 0,37 |  |
| adrenal metasases | 0,92 | -0,09 | 0,46 | -0,19 | 0,85 |  |
| bone metasases | 1,352 | 0,30 | 0,42 | 0,72 | 0,47 |  |
| brain metasases | 1,66 | 0,51 | 0,40 | 1,26 | 0,21 |  |
| hepatic metasases | 1,14 | 0,13 | 0,73 | 0,18 | 0,86 |  |
| pleural metasases | 2,11 | 0,75 | 0,65 | 1,15 | 0,25 |  |
| lung metasases | 5,07 | 1,62 | 0,82 | 1,97 | 0,05 | * |
| lymphnodes metasases | 1,21 | 0,19 | 0,76 | 0,25 | 0,81 |  |
| TTF-1 negative vs. positive | 3,25 | 1,18 | 0,47 | 2,49 | 0,01 | * |
| model 2 | HR | beta | se | z-value | p-value |  |
| ratio SUV/tumor size | 1,01 | 0,01 | 0,07 | 0,18 | 0,86 |  |
| age | 1,00 | 0,00 | 0,02 | 0,10 | 0,92 |  |
| female vs. male | 0,65 | -0,44 | 0,40 | -1,09 | 0,28 |  |
| current vs. never smoker | 1,37 | 0,32 | 0,71 | 0,45 | 0,65 |  |
| former vs. never smoker | 0,92 | -0,08 | 0,72 | -0,11 | 0,91 |  |
| unknown vs. never smoker | 1,13 | 0,12 | 0,97 | 0,12 | 0,90 |  |
| PD-L1 expression in % | 0,61 | -0,50 | 0,58 | -0,85 | 0,40 |  |
| adrenal metasases | 0,93 | -0,08 | 0,46 | -0,17 | 0,87 |  |
| bone metasases | 1,378 | 0,32 | 0,41 | 0,78 | 0,44 |  |
| brain metasases | 1,67 | 0,51 | 0,40 | 1,30 | 0,20 |  |
| hepatic metasases | 1,15 | 0,14 | 0,73 | 0,19 | 0,85 |  |
| pleural metasases | 2,23 | 0,80 | 0,64 | 1,25 | 0,21 |  |
| lung metasases | 4,93 | 1,60 | 0,83 | 1,93 | 0,05 | . |
| lymphnodes metasases | 1,19 | 0,17 | 0,76 | 0,22 | 0,82 |  |
| TTF-1 negative vs. positive | 3,20 | 1,16 | 0,47 | 2,46 | 0,01 | * |
| model 3 | HR | beta | se | z-value | p-value |  |
| MVT | 1,00 | 0,00 | 0,00 | 1,95 | 0,05 | . |
| age | 1,01 | 0,01 | 0,02 | 0,66 | 0,51 |  |
| female vs. male | 0,50 | -0,69 | 0,42 | -1,63 | 0,10 |  |
| current vs. never smoker | 0,62 | -0,47 | 0,68 | -0,70 | 0,48 |  |
| former vs. never smoker | 0,40 | -0,91 | 0,71 | -1,28 | 0,20 |  |
| unknown vs. never smoker | 0,49 | -0,71 | 0,98 | -0,72 | 0,47 |  |
| PD-L1 expression in % | 0,40 | -0,92 | 0,64 | -1,45 | 0,15 |  |
| adrenal metasases | 0,86 | -0,16 | 0,48 | -0,32 | 0,75 |  |
| bone metasases | 1,02 | 0,02 | 0,44 | 0,05 | 0,96 |  |
| brain metasases | 2,01 | 0,70 | 0,44 | 1,60 | 0,11 |  |
| hepatic metasases | 2,94 | 1,08 | 0,70 | 1,53 | 0,13 |  |
| pleural metasases | 1,38 | 0,32 | 0,69 | 0,46 | 0,64 |  |
| lung metasases | 7,51 | 2,02 | 0,81 | 2,48 | 0,01 | * |
| lymphnodes metasases | 1,45 | 0,37 | 0,79 | 0,47 | 0,64 |  |
| TTF-1 negative vs. positive | 4,16 | 1,43 | 0,50 | 2,87 | 0,004 | ** |

Abbreviations: MTV, metabolic tumor volume; PD-L1, programmed death-ligand 1; SUV, standardized uptake value; TTF-1, thyroid transcription factor 1.

**Table 6. Comparison of prognostic model performance for OS and PFS**

Abbreviations: AIC, Akaike Information Criterion; BIC, Bayesian Information Criterion; C-Index, Harrell’s concordance index; EPV, events per variable; OS, overall survival; PET, positron emission tomography, PFS, progression-free survival; SE, standard error.

**Table 7. Likelihood ratio test comparison of nested prognostic models for overall survival and progression-free survival**

Abbreviations: Chisq, chi-square statistic; df, degrees of freedom; FDR_p, p-value adjusted for multiple testing using the Benjamini–Hochberg method; loglik, log-likelihood; n_full, number of patients in the full model; n_reduced, number of patients in the reduced model; OS, overall survival; PFS, progression-free survival.

**Supplementary Table 1. Baseline characteristics according to availability of MTV measurements.**

**Supplementary Table 2. Interaction effects between PET-derived parameters and inflammatory biomarkers on OS and PFS**

Abbreviations: CI, confidence interval; CRP, C-reactive protein; FDR, false discovery rate adjusted using the Benjamini–Hochberg procedure; HR, hazard ratio; LDH, lactate dehydrogenase; MTV, metabolic tumor volume; NLR, neutrophil-to-lymphocyte ratio; OS, overall survival; PD-L1, programmed death-ligand 1; PFS, progression-free survival; SUV, standardized uptake value.

**Supplementary Table 3. Schoenfeld residual tests for the proportional hazards assumption in the combined models for OS and PFS**

Abbreviations: Chisq, chi-square statistic; df, degrees of freedom; OS, overall survival; PFS, progression-free survival; PD-L1, programmed death-ligand 1; TTF-1, thyroid transcription factor 1.

**Supplementary Table 4. Variables selected by LASSO penalized Cox regression models for OS and PFS**

Abbreviations: ANZ_met, adrenal metastases; CRP, C-reactive protein; LASSO, least absolute shrinkage and selection operator; MTV, metabolic tumor volume; OS, overall survival; PFS, progression-free survival; TTF-1, thyroid transcription factor 1; λ₁se, regularization parameter selected according to the one-standard-error rule; λmin, regularization parameter minimizing cross-validation error.

**Supplementary Table 5. Missing data across clinical, laboratory, and imaging variables included in the analysis**

Abbreviations: CRP, C-reactive protein; LDH, lactate dehydrogenase; MTV, metabolic tumor volume; NLR, neutrophil-to-lymphocyte ratio; PD-L1, programmed death-ligand 1; TTF-1, thyroid transcription factor 1.

**Supplementary Table 6. Internal validation of the combined prognostic model using bootstrap resampling**

Abbreviations: ANZ_met, adrenal metastases; CRP, C-reactive protein; LDH, lactate dehydrogenase; MTV, metabolic tumor volume; NLR, neutrophil-to-lymphocyte ratio; OS, overall survival; PD-L1, programmed death-ligand 1; PFS, progression-free survival; SD, standard deviation; SUV, standardized uptake value.

**Supplementary Table 7. Treatment distribution**

| Treatment distribution | N |
| --- | --- |
| Chemo-ICI | 55 |
| ICI alone | 16 |
| Treatment exact distribution |  |
| Atezolizumab | 2 |
| Carboplatin/Pemetrexed/Nivolumab/Ipilimumab | 1 |
| Carboplatin/Pemetrexed/Pembrolizumab | 43 |
| Carboplatin/Paclitaxel/Pembrolizumab | 3 |
| Cisplatin/Pemetrexed/Pembrolizumab | 8 |
| Pembrolizumab | 14 |
|  |  |

**Supplementary Table 8. Exploratory stratified multivariable Cox regression models for overall survival and progression-free survival in the MTV cohort.**

**Supplementary Table 9. Exploratory interaction analyses between PET biomarkers and PD-L1 expression**

**Supplementary Table 10. Exploratory restricted cubic spline analyses for PET biomarkers**
